# Continuous-Flow Synthesis of *N*-Succinimidyl 4-[^18^F]fluorobenzoate Using a Single Microfluidic Chip

**DOI:** 10.1371/journal.pone.0159303

**Published:** 2016-07-13

**Authors:** Hiroyuki Kimura, Kenji Tomatsu, Hidekazu Saiki, Kenji Arimitsu, Masahiro Ono, Hidekazu Kawashima, Ren Iwata, Hiroaki Nakanishi, Eiichi Ozeki, Yuji Kuge, Hideo Saji

**Affiliations:** 1 Graduate School of Pharmaceutical Sciences, Kyoto University, Kyoto, Kyoto, Japan; 2 Department of Analytical and Bioinorganic Chemistry, Kyoto Pharmaceutical University, Kyoto, Kyoto, Japan; 3 Technology Research Laboratory, Shimadzu Corporation, Souraku-gun, Kyoto, Japan; 4 School of Pharmacy and Pharmaceutical Sciences, Mukogawa Women’s University, Nishinomiya, Hyogo, Japan; 5 Radioisotope Research Center, Kyoto Pharmaceutical University, Kyoto, Kyoto, Japan; 6 CYRIC, Tohoku University, Sendai, Miyagi, Japan; 7 Central Institute of Isotope Science, Hokkaido University, Sapporo, Hokkaido, Japan; Wayne State University, UNITED STATES

## Abstract

In the field of positron emission tomography (PET) radiochemistry, compact microreactors provide reliable and reproducible synthesis methods that reduce the use of expensive precursors for radiolabeling and make effective use of the limited space in a hot cell. To develop more compact microreactors for radiosynthesis of ^18^F-labeled compounds required for the multistep procedure, we attempted radiosynthesis of *N*-succinimidyl 4-[^18^F]fluorobenzoate ([^18^F]SFB) via a three-step procedure using a microreactor. We examined individual steps for [^18^F]SFB using a batch reactor and microreactor and developed a new continuous-flow synthetic method with a single microfluidic chip to achieve rapid and efficient radiosynthesis of [^18^F]SFB. In the synthesis of [^18^F]SFB using this continuous-flow method, the three-step reaction was successfully completed within 6.5 min and the radiochemical yield was 64 ± 2% (*n* = 5). In addition, it was shown that the quality of [^18^F]SFB synthesized on this method was equal to that synthesized by conventional methods using a batch reactor in the radiolabeling of bovine serum albumin with [^18^F]SFB.

## Introduction

Positron emission tomography (PET) [1] is a powerful non-invasive imaging technology for investigating physiological parameters in living human and animal whole bodies using molecular probes labeled with PET radioisotopes. The development and preparation of such radiolabeled molecules is a major synthetic challenge because of the short half-lives of positron-emitting radionuclides such as ^18^F, ^11^C, ^13^N, and ^15^O.

Microfluidic technologies have many advantages for the rapid synthesis of short-lived radiopharmaceuticals for PET. A microreactor is a synthesizer that allows reactions to occur inside microchannels at the nanoliter/microliter scale. The large specific surface area enables effective heat conduction, and the short diffusion distances reduce mixing times. The short reaction times and good chemical yields [2] achieved using microreactors have attracted a high level of interest in PET radiochemistry. Microreactors are also suitable for handling and reacting small amounts of reagents and are therefore appropriate for the synthesis of expensive precursors or tracer compounds, which may be in limited supply [3–7]. Furthermore, radiochemical reactions performed on a compact microfluidic chip can be easily shielded. A compact microfluidic chip does not require the space and resources required by conventional synthesizers [8–10].

The use of microreactors in single-step radiolabeling (e.g., ^18^F and ^11^C) [11–14] and the production of PET probes [15–24] have been reported. Many recently developed PET probes involve lengthy, multistep, and inefficient synthetic procedures because the parent skeleton of probes has become diversified. Therefore, the development of a microreactor that can perform a multistep synthesis is desirable.

**Fig 1:**
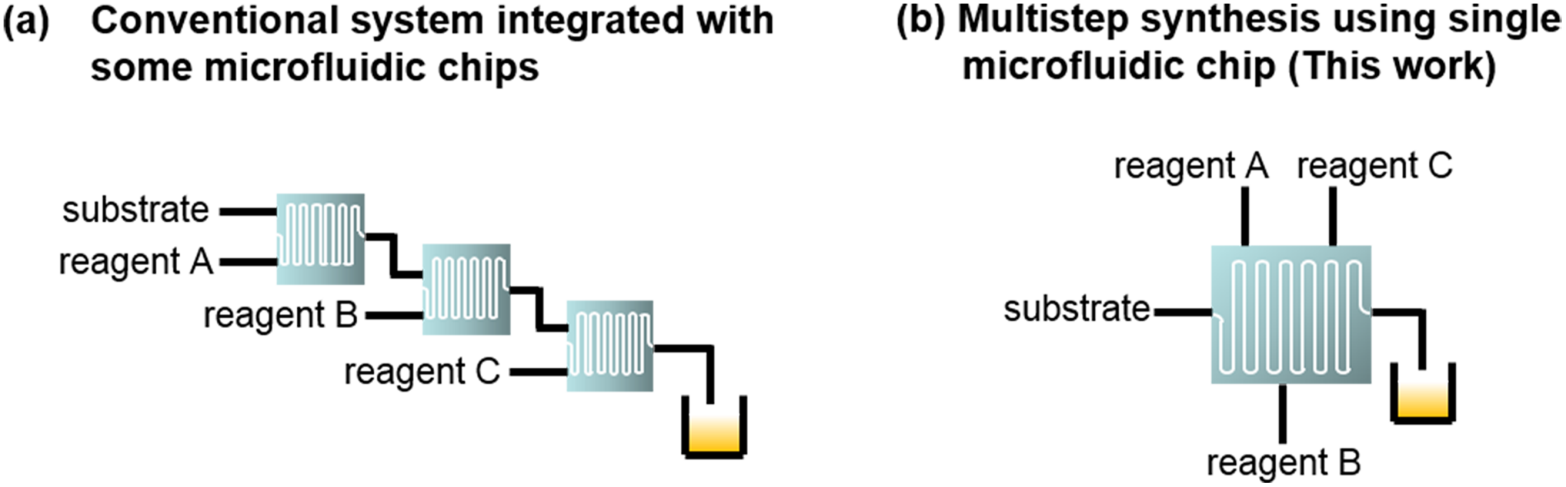
Concepts for microfluidic syntheses that require multistep reactions.

Two types of approaches, continuous-flow and batch-mode microreactors, have been attempted for multistep synthesis of radiopharmaceuticals. In case of continuous-flow systems, several microreactor-included microfluidic chips have been used (Fig 1a) [17–28]. However, these systems have the following disadvantages: 1) multiple microfluidic chips are necessary, 2) there are large dead volumes between the microfluidic chips, and 3) the control systems are complicated. To address these problems, we have attempted to develop a continuous-flow synthetic method in a multistep reaction using a single microfluidic chip (Fig 1b).

**Fig 2:**
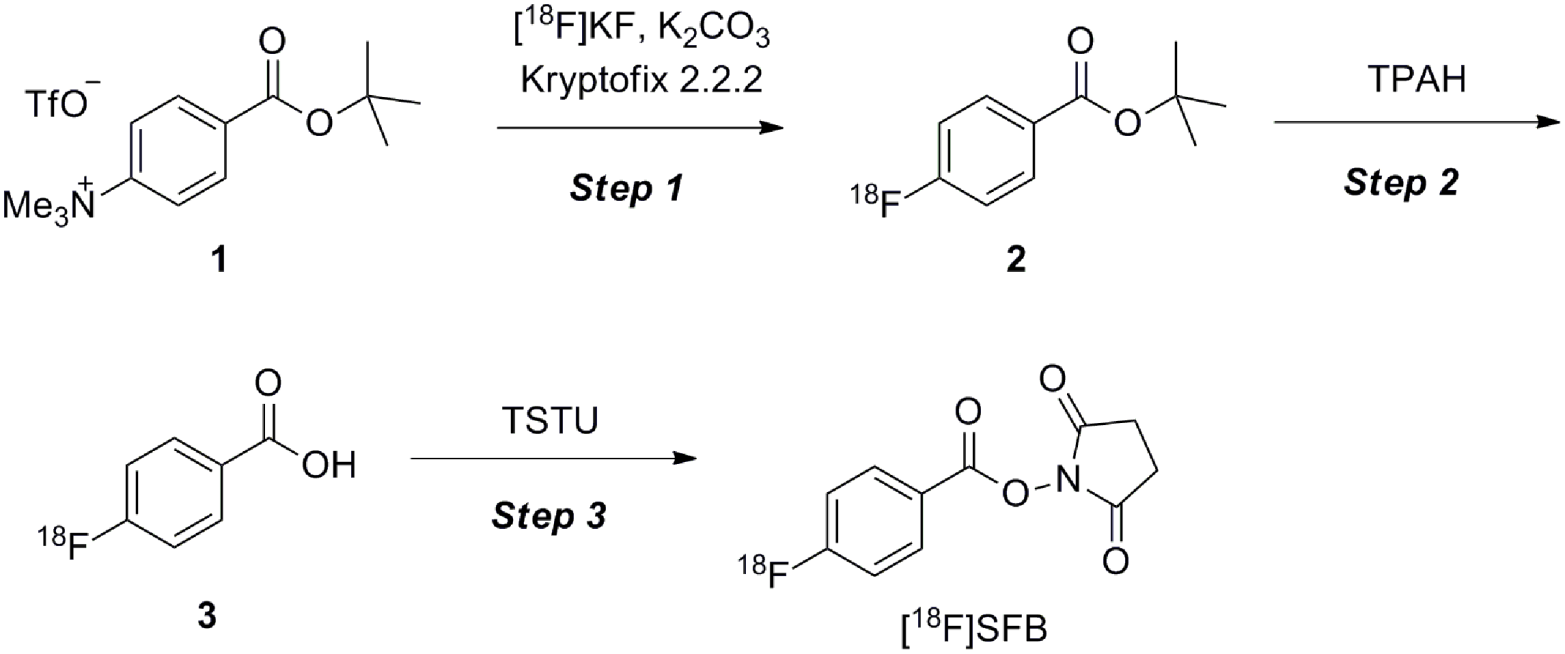
Synthetic method for [^18^F]SFB.

Many clinical PET probes incorporate ^18^F. From a PET synthetic perspective, the half-life of ^18^F is sufficiently long to enable multistep synthesis. The radiosynthesis of *N*-succinimidyl 4-[^18^F]fluorobenzoate ([^18^F]SFB) [29–36] was selected as the model reaction for multistep synthesis evaluation because [^18^F]SFB is a well-known prosthetic group for peptides, proteins, and antibodies in PET radiochemistry. The synthesis of [^18^F]SFB requires the following three steps: 1) [^18^F]fluorination of an aromatic precursor, i.e., 4-(*tert*-butoxycarbonyl)-*N*,*N*,*N*-trimethylphenylammonium triflate (**1**) [31], 2) hydrolysis of *tert*-butyl 4-[^18^F]fluorobenzoate (**2**) with tetrapropylammonium hydroxide (TPAH), and 3) succinimidylation of the 4-[^18^F]fluorobenzoic acid (**3**) with *O*-(*N*-succinimidyl)-*N*,*N*,*N'*,*N'*-tetramethyluronium tetrafluoroborate (TSTU) [30,31] (Fig 2). To date, attempts have been made to reduce the time required to synthesize [^18^F]SFB and to improve the yields using modified commercial synthesizers and microwave synthetic technology; [34–36] however, further improvements are desired.

## Materials and Methods

### General Experimental

Analytical and preparative high-performance liquid chromatography (HPLC) was performed using a Shimadzu LC-20AD (Shimadzu Corporation, Kyoto, Japan) solvent delivery system at a flow rate of 1.0 mL/min an with SPD-10AP (Shimadzu Corporation) UV detector (254 nm) and Aloka NDW-351D (Hitachi Aloka Medical, Ltd., Tokyo, Japan) RI detector. A Cosmosil 5C_18_-PAQ column (4.6 × 150 mm, Nacalai Tesque Inc., Kyoto, Japan) was used for reverse-phase HPLC. Conditions for HPLC are defined as follows: condition 1) MeCN/water = 55/45; condition 2) MeCN:ammonium acetate buffer (50 mM, pH 4.0) = 25:75 (over 7 min), gradually adjusted to 80:20 (over 8 min), and followed by 80:20; condition 3) MeCN:ammonium acetate buffer (50 mM, pH 4.0) = initiated from 25:75, gradually adjusted to 80:20 (over 20 min), and followed by 80:20. A part of the reaction mixture was purified by HPLC to give the solution of ^18^F-labeled products. From the radioactivity of the resulting solution, the decay-corrected conversion yields were calculated with the following formula: conversion yield (%) = (decay-corrected radioactivity of ^18^F-labeled products obtained by radio-HPLC purification/radioactivity of ^18^F loaded into the radio-HPLC) × 100. The radioactivity of ^18^F was measured using a curiemeter (IGC-3, Aloka) or NaI (Tl) gamma scintillation counter (Cobra Auto-Gamma Counter 5003, Perkin-Elmer (Packard), Waltham, MA, USA). A screw-cap pressure vessel was used as a batch reactor (purchased from Oofuna, Ltd., Osaka, Japan) [37]. Most chemicals were reagent-grade, purchased from Merck (Darmstadt, Germany) and Sigma-Aldrich (St. Louis, MO, USA), and used without further purification. Precursor **1** and *N*-succinimidyl fluorobenzoate (non-radioactive SFB) [31] were synthesized according to a previously published procedure [30,31].

### Preparation of [^18^F]fluoride ion

[^18^F]Fluoride ion was produced using a cyclotron (CYPRIS HM-18, Sumitomo Heavy Industries, Tokyo, Japan) via an ^18^O(p,n)^18^F reaction and passed through a Sep-Pak Light QMA cartridge (Waters Corporation, Milford, MA, USA) as an aqueous solution in ^18^O-enriched water. The cartridge was dried in an air-flow, and the ^18^F-active material was eluted with an aqueous solution of K_2_CO_3_ (33 mM, 0.5 mL).

### [^18^F]Fluorination (step 1) using batch reactor

An aqueous solution of ^18^F^−^ (ca. 37 MBq, 100–500 μL, containing 33 mM K_2_CO_3_) was added to a solution of Kryptofix 2.2.2 (10 mg) in MeCN (500 μL). The solvent was removed under a stream of argon gas at 110°C. Azeotropic drying was repeated three times with 400 μL portions of MeCN to give full dehydration. A solution of **1** (1 mg) in DMSO (1 mL) was added to the mixture, and the reaction was heated at 120°C for 1, 3, 5, 10, or 15 min. The reaction was quenched by the mixture solution of DMSO and water (50/50). The mixture was purified by preparative radio-HPLC (condition 1) to give a solution of pure **2** (Rt = 11.0 min). The radioactivity of ^18^F was determined using an NaI (Tl) gamma scintillation counter. Data are the mean ± S.D. (*n* = 5) decay corrected.

### Hydrolysis of 3 (step 2) using batch reactor

An aqueous solution of TPAH (1 M, 4 μL) was diluted with DMSO (96 μL), and the solution was added to a solution of **2** (prepared by step 1) in DMSO (100 μL). The mixture was heated at 120°C for 5, 30, and 60 s. The reaction was quenched by an aqueous solution of 0.5% acetic acid, and the mixture was purified by preparative radio-HPLC (condition 2) to give a solution of pure **3** (Rt = 10.7 min). The radioactivity of ^18^F was determined using an NaI (Tl) gamma scintillation counter. Data are the mean ± S.D. (*n* = 4) decay corrected.

### Microreactor system

**Fig 3:**
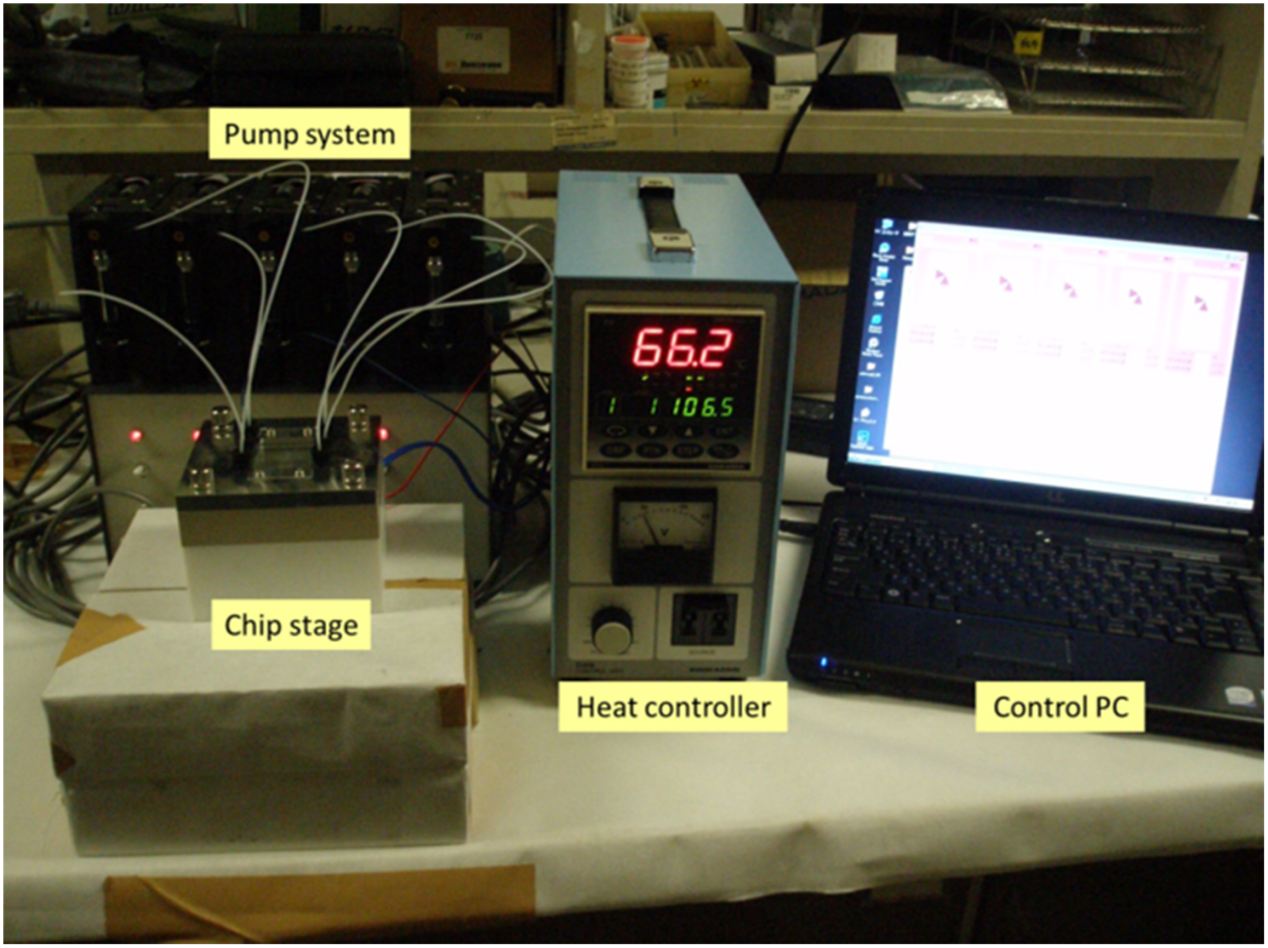
Microreactor system developed in this study.

The microreactor system developed in this study, including the pump system, chip stage, heat controller, and operating soft, was manufactured by Shimadzu Corporation. The jig used to secure the microfluidic chip was placed onto a heating plate for the reaction, and the temperature was controlled using feedback from a laminated sensor on the chip (Fig 3).

### Microfluidic chip designed for single-step reaction (chip 1)

**Fig 4:**
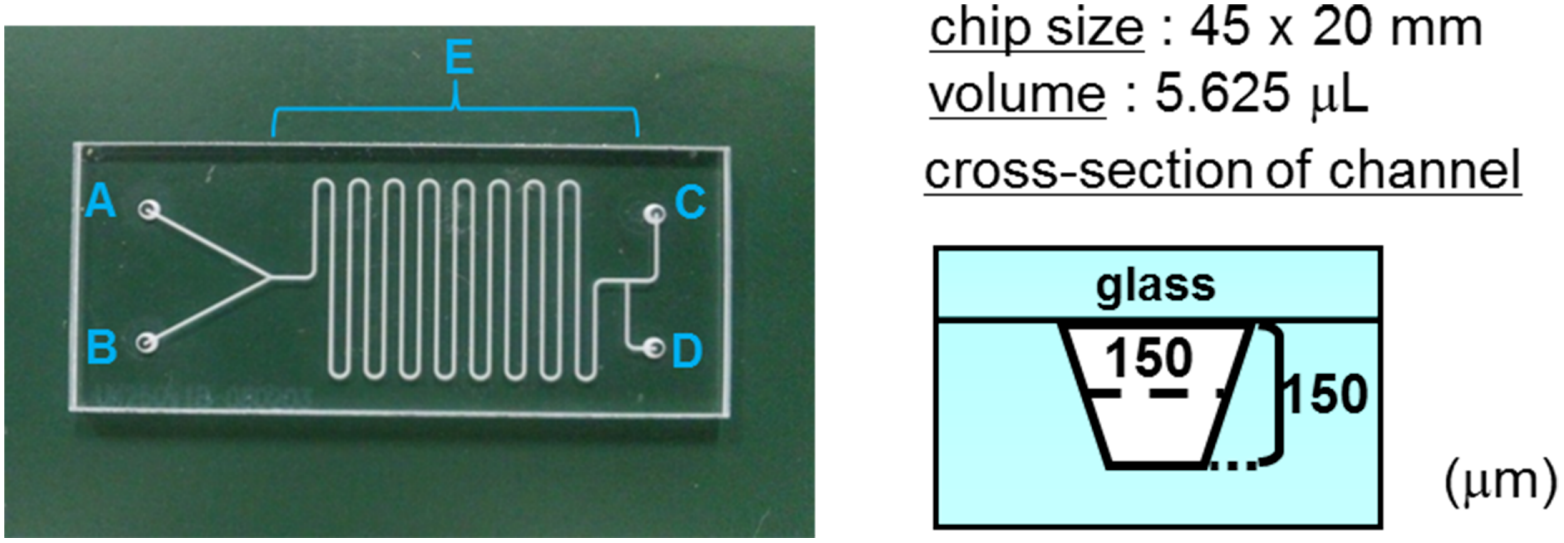
Design of chip 1 for a single reaction.

Chip 1 was formed by bonding two glass substrates. Reaction channel **E**, reagent injection ports (inlet **A**, **B**), a liquid discharge port (outlet **D**), and a reaction-stop reagent injection port (inlet **C**) to channel **E** were formed. The channels and through holes were formed by sandblasting, and the glass substrates were bonded using hydrofluoric acid. The width of reaction channel **E** and the connection channels was 150 μm, and the depth was 150 μm. The length of reaction channel **E** was 250 mm, and the volumetric capacity was 5.625 μL (Fig 4). In addition, the surface temperature of chip 1 placed on heating plate was monitored at several points using a data logger (NR-1000, Keyence, Osaka, Japan) and thermograph (Thermo-Tracer 6T61, NEC San-ei, Tokyo, Japan).

#### Diffusional efficiency in microfluidic chip (for chip 1)

To evaluate the diffusional efficiency in our microfluidic chip, both DMSO and black-colored DMSO were introduced into chip 1 (designed for single-step reaction) through inlet **A** and **B**, respectively. The total flow rate was 33.76 μL/min (residence time: 10 s). When the chip was heated to 120°C on a heating plate, the solution state at three points of the flow-channel was observed by a stereomicroscope (STZ-168-TL, Shimadzu Corporation, Kyoto, Japan).

### [^18^F]Fluorination (step 1) using microreactor

A solution of **1** (1 mg) in DMSO (500 μL) was introduced into the chip 1 through inlet **A**. An aqueous solution of ^18^F^−^ (ca. 37 MBq, 100–500 μL, containing 33 mM K_2_CO_3_) and a solution of Kryptofix 2.2.2 (10 mg) in MeCN (500 μL) were azeotropically dehydrated in the same manner as the above dehydration. Following this, the mixture was dissolved in DMSO (500 μL) and introduced through inlet **B**. The reaction was performed on chip 1 heated at 120°C. A mixture solution of DMSO and water (80/20) was introduced through inlet **C** as a quenching reagent. The reaction mixture collected from outlet **D** was purified in the same manner as that for the bacth reactor. The radioactivity of ^18^F was determined using a NaI (Tl) gamma scintillation counter. Data are the mean ± S.D. (*n* = 5) decay corrected.

### Hydrolysis of 3 (step 2) using microreactor

A solution of **2** (prepared by step 1) in DMSO (100 μL) was introduced into chip 1 through inlet **A**. An aqueous solution of TPAH (1 M, 4 μL) was diluted with DMSO (96 μL), and the solution was introduced through inlet **B**. An aqueous solution of 0.5% acetic acid was injected through inlet **C** to quench the reaction.

The reaction mixture collected from outlet **D** was purified in the same manner as that for the batch reactor to give a solution of pure **3** (Rt = 10.7 min). The radioactivity of ^18^F was determined using a NaI (Tl) gamma scintillation counter. Data are the mean ± S.D. (*n* = 4) decay corrected.

### Evaluation of effect of water content for succinimidylation (step 3) (using batch reactor)

The mixture of ^18^F^−^ (ca. 37 MBq, 100–500 μL, containing 33 mM K_2_CO_3_) and Kryptofix 2.2.2 (10 mg) in MeCN (500 μL) was azeotropically dehydrated in the same manner as the above dehydration. A solution of precursor **1** (5 mg) in MeCN (1 mL) was added to the mixture, and the reaction was performed at 90°C for 10 min. An aqueous solution of TPAH (1 M, 20 μL) was added, and the reaction was performed at 120°C for 5 min to give intermediate **3**. After the reaction mixture was azeotropically dehydrated with MeCN, the mixture was dissolved in MeCN and reacted with TSTU to synthesize [^18^F]SFB. The reaction solutions were also adjusted to 1.6%–51% of water content and reacted with TSTU. The mixture was purified by preparative HPLC (condition 3) to give a solution of pure [^18^F]SFB (Rt = 11.0 min). The radioactivity of ^18^F was determined using a NaI (Tl) gamma scintillation counter. Data are the mean ± S.D. (*n* = 5) decay corrected.

### Microfluidic chip designed for three-step reaction (chip 2)

**Fig 5:**
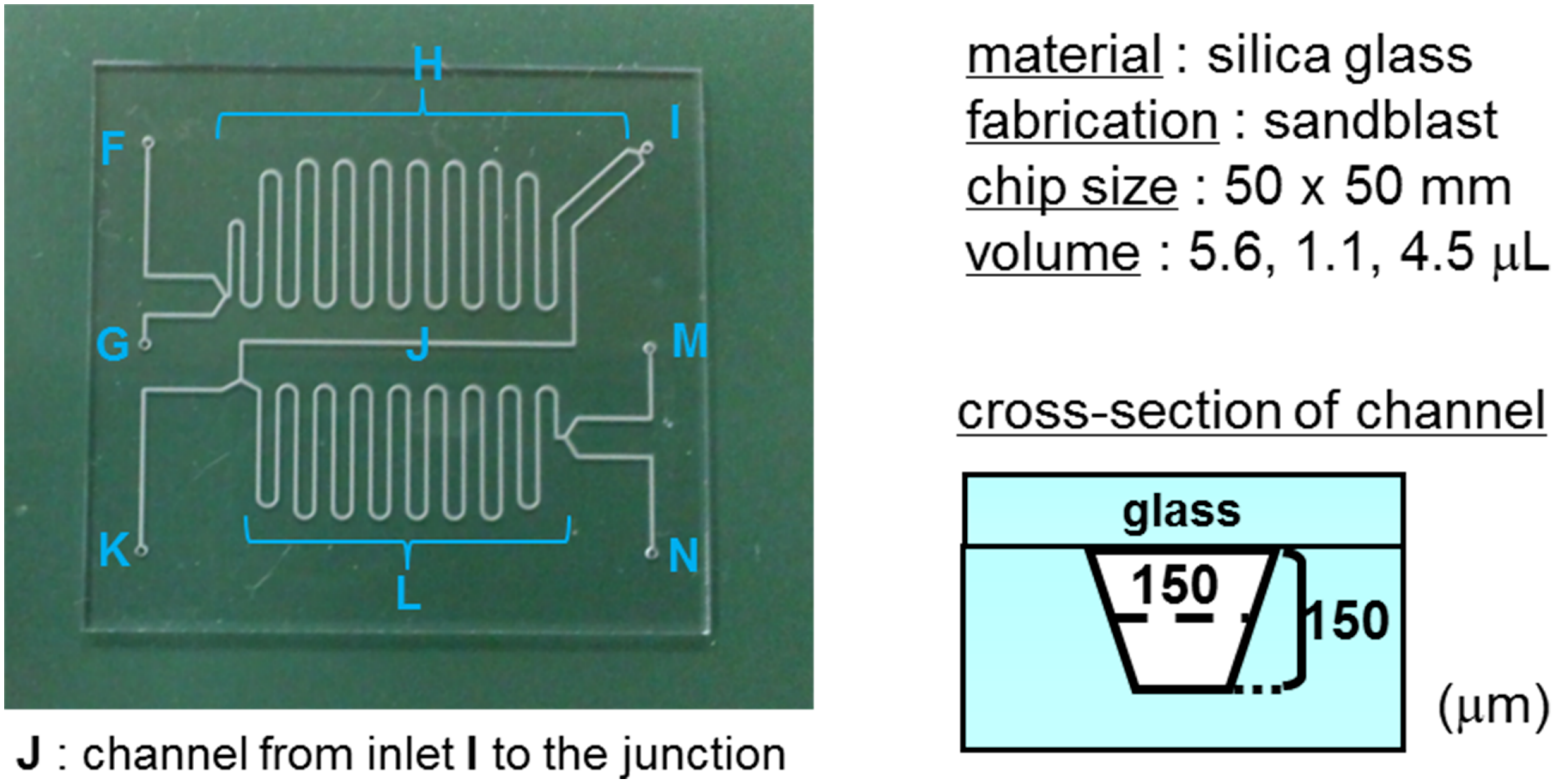
Design of chip 2 for a three-step reaction.

Chip 2 was formed by bonding two glass substrates. Reaction channels (**H**, **J**, and **L**) and channels for connecting the raw material injection port, a liquid discharge port (outlet **M**), a quenching reagent injection port (inlet **N**), and injection ports (inlet **F**, **G**, **I**, and **K**) to inject various reagents into the reaction channel were formed. The channels and through holes were formed by sandblasting, and the glass substrates were bonded using hydrofluoric acid. The width of the reaction channels **H**, **J**, and **L**, and the connection channels was 150 μm, and the depth was 150 μm. The length of the first-step reaction channel **H** was 250 mm and its volumetric capacity was 5.625 μL. The length of the second-step reaction channel **J** was 50 mm and its volumetric capacity was 1.125 μL. The length of the third-step reaction channel **L** was 200 mm, and its volumetric capacity was 4.5 μL (Fig 5). As is the case in chip 1, the surface temperature of chip 2 placed on heating plate was monitored at several points using a data logger and thermograph.

### Continuous-flow synthesis of [^18^F]SFB using a microreactor

Kryptofix 2.2.2 (10 mg) and ^18^F^−^ (ca. 37 MBq) were azeotropically dehydrated, dissolved in DMSO (500 μL), and introduced into chip 2 through inlet **A** at flow rate of 0.56 μL/min. A solution of **1** (1 mg) in DMSO (500 μL) was set on inlet **B** and introduced at flow rate of 0.56 μL/min. A diluted solution of TPAH (1 M aqueous solution, 40 μL) with DMSO (500 μL) and a solution of TSTU (15 mg) in DMSO (500 μL) were set on inlet **I** and **K** and then introduced at flow rates of 1.12 and 2.24 μL/min, respectively. The reaction was performed on chip 2 heated at 120°C, and reaction times of step 1, 2, and 3 were 5 min, 30 s, and 1 min, respectively. DMSO/water (90/10) was introduced through inlet **N** as a quenching reagent.

The mixture was purified by preparative HPLC (condition 3) to give a solution of pure [^18^F]SFB (Rt = 11.0 min). The radioactivity of ^18^F was determined using a NaI (Tl) gamma scintillation counter. Data are the mean ± S.D. (*n* = 5) decay corrected.

### Radiolabeling of BSA with [^18^F]SFB

After the synthesis of [^18^F]SFB using chip 2, the overall mixture collected from outlet **M** was diluted with 5% acetic acid and applied to a Sep-Pak Accell Plus CM Cartridge (Waters Corporation). A Sep-Pak PS-2 Plus Cartridge (Waters Corporation) was attached to the Sep-Pak Accell Plus CM Cartridge, and the cartridges were washed with 20 mL of MeCN/water (20/80). After flowing N_2_ gas for 10 s, the Sep-Pak Accell Plus CM Cartridge was removed. [^18^F]SFB was eluted from the Sep-Pak Plus PS-2 Cartridge with 2.0 mL of MeCN. The isolated radiochemical yield was measured using a curiemeter. MeCN was removed under a stream of argon at 50°C. After drying, [^18^F]SFB was dissolved in sodium borate buffer solution (0.05 M, pH 8.5: solution I). BSA was dissolved in sodium borate buffer solution (0.05 M, pH 8.5, 20 mg/mL: solution II). For the microreactor reaction, solution I and II were run into inlets **A** and **B** on chip 1 at a flow rate of 0.56 μL/min at 37°C. The reaction was stopped by adding 0.01 M HCl solution. The reaction mixture was analyzed using the electrophoresis (0.8 mA/cm, 45 min). The position of radiolabeled BSA was previously confirmed by Ponceau staining. The filter paper was then cut equally. The radioactivity was measured by an NaI (Tl) gamma scintillation counter, and the conversion yield was calculated from the radioactivity of [^18^F]SFB. Data are the mean ± S.D. (*n* = 4) decay corrected.

## Results and Discussion

**Fig 6:**
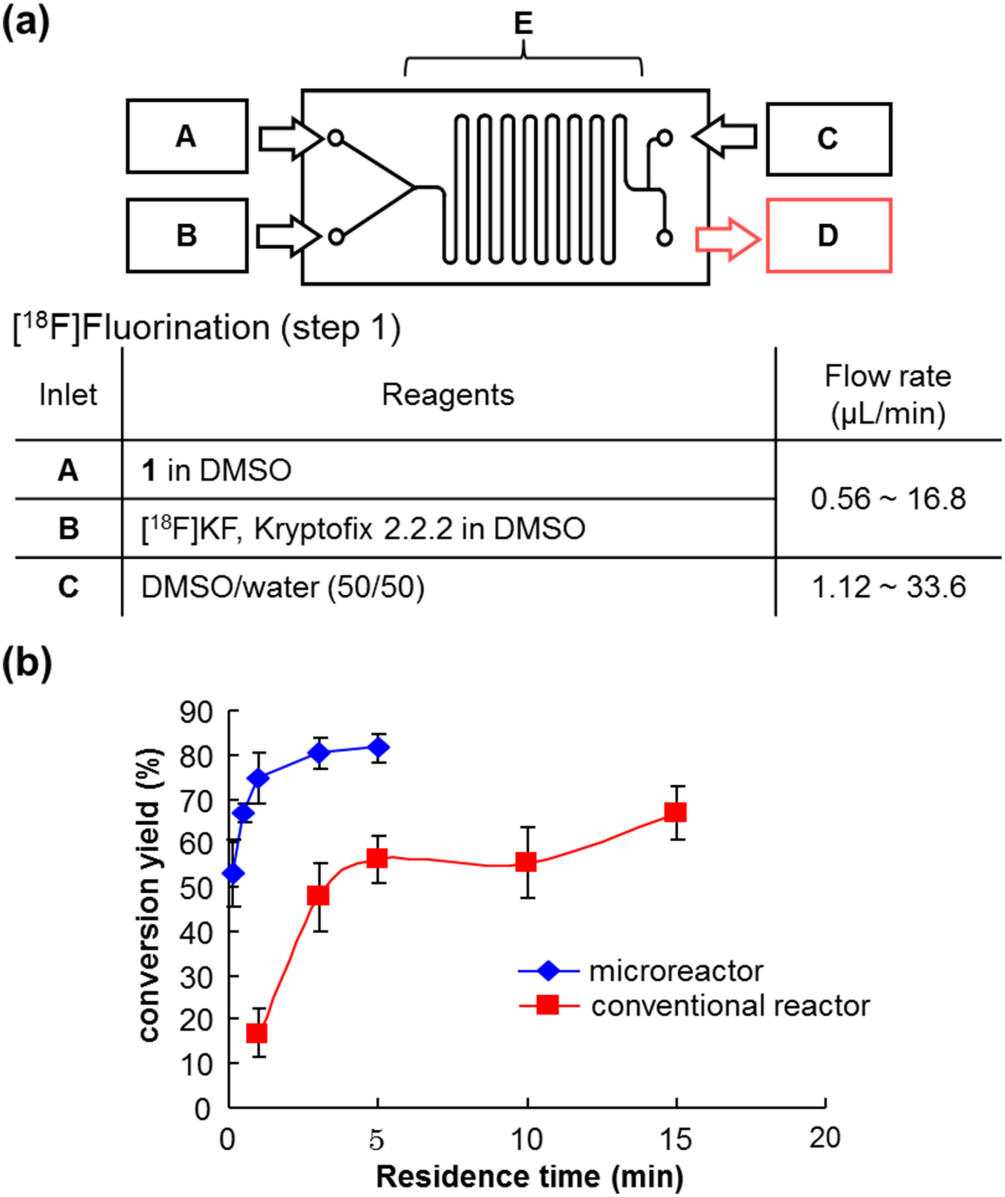
(a) Procedure for [^18^F]fluorination (step 1) using microreactor with chip 1. (b) Comparison with microreactor (data are the mean ± S.D., *n* = 5) and batch reactor (data are the mean ± S.D., *n* = 4).

To apply the three-step reaction of [^18^F]SFB to a continuous-flow synthetic method using a single microfluidic chip, we initially examined ^18^F-fluorination, which is the first step of radiosynthesis of [^18^F]SFB (Fig 2). A solution of **1** in dimethyl sulfoxide (DMSO) was run into inlet A, and a mixture of [^18^F]KF and Kryptofix 2.2.2 in DMSO was run into inlet **B** on the designed microfluidic chip (chip 1) pre-heated at 120°C (Fig 6a). After passing channel E, which is regarded as a reaction field, the mixture was quenched by the solvent injected through inlet **C**. The optimal reaction time (residence time) was established by the change in the flow rate. The conversion yield of **2** reached 75 ± 6% (*n* = 5) in less than 1 min and 81 ± 3% (*n* = 5) at 5 min. The use of a batch reactor under oil bath heating condition gave a conversion of 56 ± 5% (*n* = 4) at 5 min and 67 ± 6% (*n* = 4) at 15 min (Fig 6b). The conversion yield was higher with the microreactor at 5 min than with the batch reactor at 15 min.

**Fig 7:**
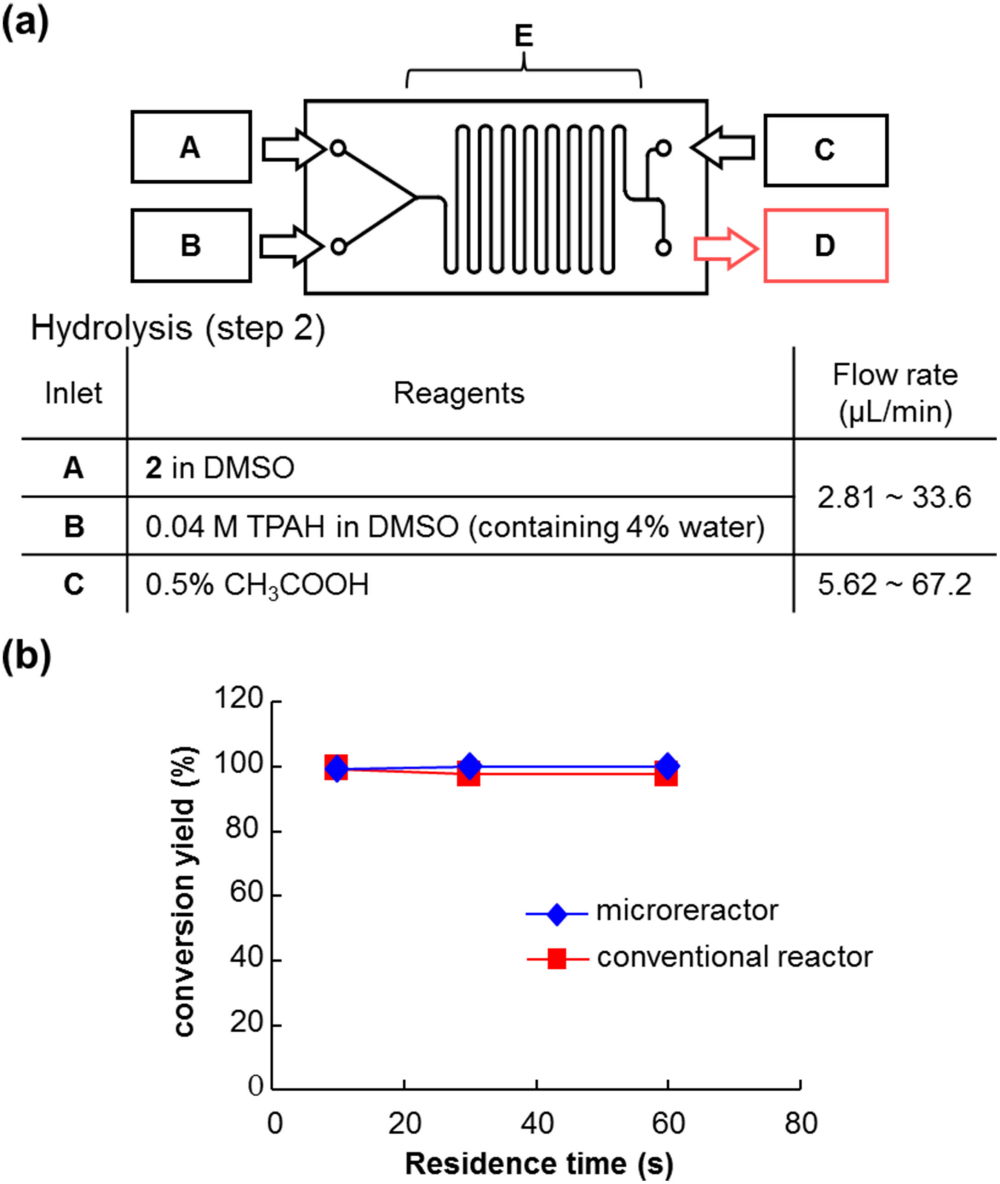
(a) Procedure for hydrolysis (step 2) using microreactor with chip 1. (b) Comparison with microreactor (data are the mean ± S.D., *n* = 4) and batch reactor (data are the mean ± S.D., *n* = 4).

Following this, the effectiveness of the microreactor for hydrolysis of ester **2**, which was prepared using a batch reactor, was examined (Fig 7a). Treatment of **2** with an aqueous solution of TPAH in DMSO on chip 1 gave **3** rapidly and quantitatively. Fig 7b shows that there was little difference between the reaction using the microreactor and the batch reactor. This result suggests that the hydrolysis of **2** progresses easily.

**Fig 8:**
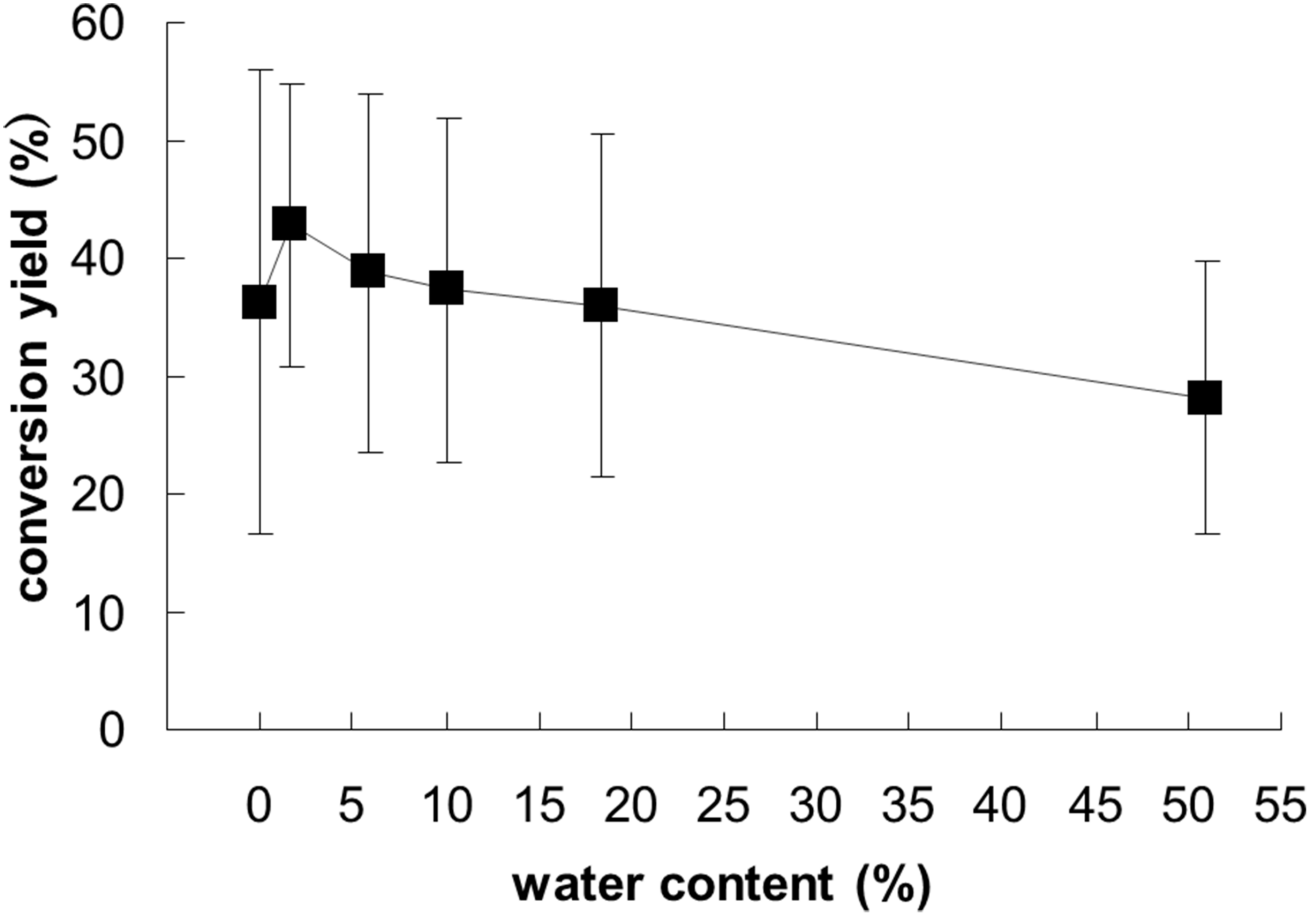
Radiochemical yield of [^18^F]SFB by one-pot radiosynthesis under different water content conditions (data are the mean ± S.D., *n* = 3).

We then attempted to examine succinimidylation (step 3) using the microreactor. However, [^18^F]**2** was difficult to isolate because of its high volatility. Hence, we investigated the succinimidylation of **3** without any treatment after hydrolysis. In one-pot radiosynthesis of [^18^F]SFB using a batch reactor, the mixture obtained after hydrolysis is azeotropically dehydrated and then allowed to react with TSTU to give [^18^F]SFB [34]. Bannwarth et al. have reported that succinimidylation with only a slight excess of TSTU is rapid and clean, even in the presence of water [38]. To apply continuous-flow synthesis using a microreactor, we investigated simplification of the procedure by omitting the dehydration process. In our synthetic strategy, a solution of TPAH in DMSO involving 4% water was used in the above hydrolysis process using a microreactor. Therefore, when the solution of TPAH in DMSO is used without any treatment for succinimidylation, the water content is 2%. To evaluate the effect of the water content on succinimidylation, one-pot radiosynthesis of [^18^F]SFB was performed using a batch reactor. Acid **3** was prepared using a previously reported method [34] and then reacted with TSTU in MeCN containing 1.6% to 51% water at 90°C. The conversion yield of [^18^F]SFB did not decrease significantly even when the water content of the reaction solution was as much as 50% (Fig 8). Therefore, the azeotropic dehydration step after hydrolysis could be omitted under our synthetic conditions.

**Fig 9:**
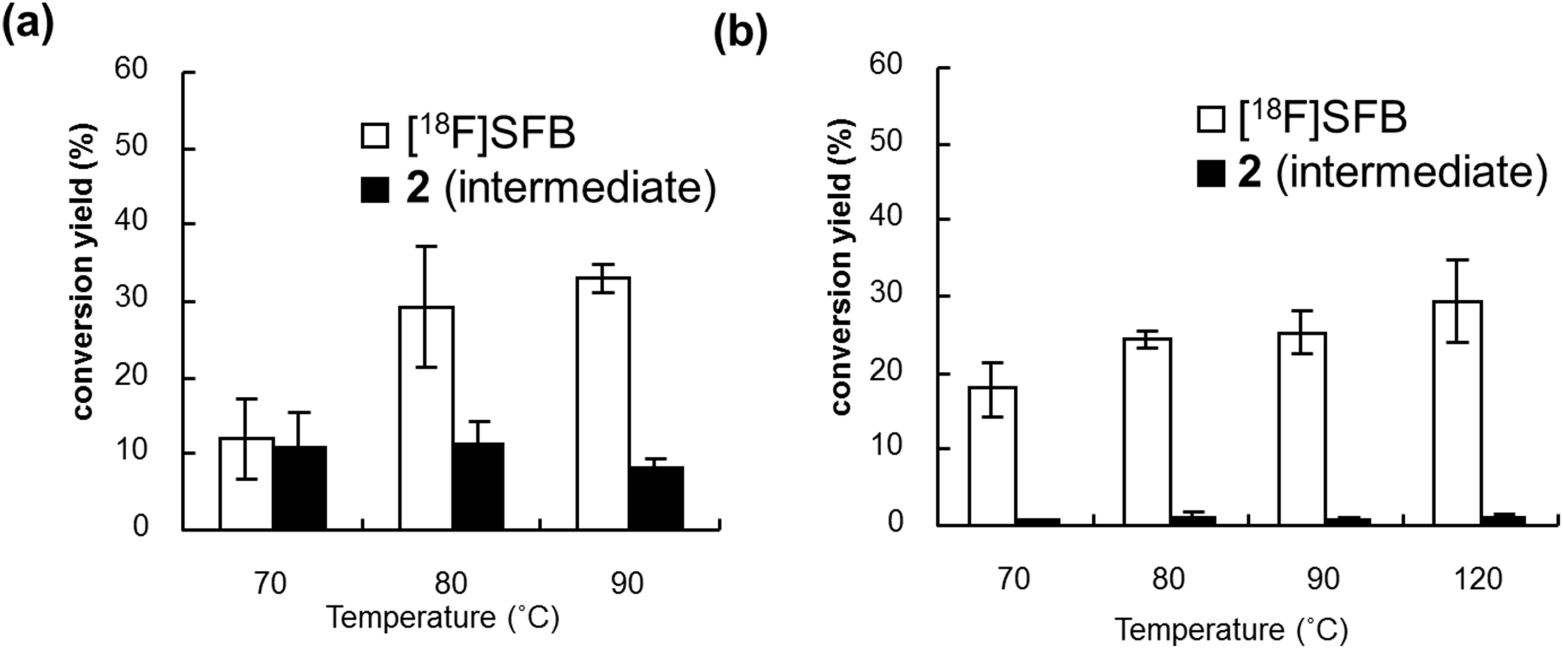
One-pot synthesis of [^18^F]SFB in (a) MeCN or (b) DMSO using a batch reactor (data are the mean ± S.D., *n* = 3).

To simplify the system, it is preferable if the reaction temperature in a multistep reaction run with a single microfluidic chip is the same for all steps. Thus, the reaction temperatures for the three-step reaction were standardized using one-pot synthesis of [^18^F]SFB in a batch reactor with either acetonitrile (MeCN) or DMSO as the solvent. Reaction times of 10, 5, and 2 min were set for [^18^F]fluorination, hydrolysis, and succinimidylation, respectively, and the corresponding reagents were successively added to a solution of **1** in MeCN at three reaction temperatures, i.e., 70, 80, and 90°C, respectively (Fig 9a). [^18^F]SFB was obtained with 33% of conversion yield at 90°C using a one-pot procedure; in addition, the residual intermediate ester **2** was observed at 8%. It was confirmed that at temperatures greater than 100°C, MeCN began to leak at the joint with the microfluidic chip in the microreactor. When DMSO was used, four reaction temperatures, i.e., 70, 80, 90, and 120°C, were tested (Fig 9b). [^18^F]SFB was obtained with 29% of coversion yield at 120°C and the amount of residual **2** was less (<1%) at all temperatures, and the production of [^18^F]SFB was promoted at higher temperatures. It was confirmed that DMSO began to leak at the joint at temperatures greater than 120°C. Therefore, we chose DMSO as the solvent and 120°C as the set temperature.

**Fig 10:**
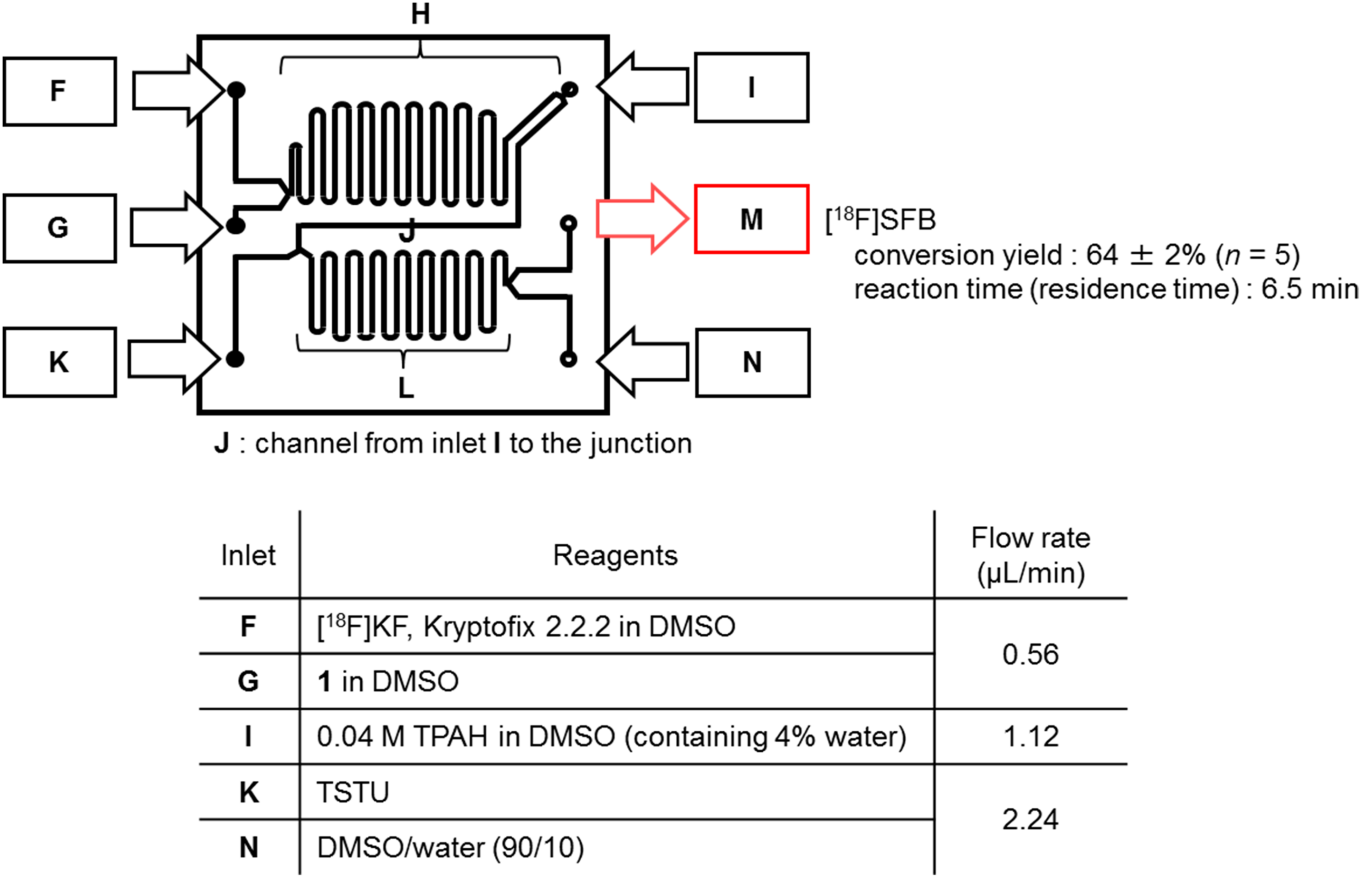
(a) Microfluidic chip for three-step reaction (chip 2) and (b) conditions for continuous-flow synthesis of [^18^F]SFB.

Based on the results, we designed a 50-mm × 50-mm microfluidic chip (chip 2) with a total flow channel of length 500 mm (Fig 5) for the three-step reaction. Chip 2 had five inlets along the entire channel and one outlet. The channel lengths were 250 mm for step 1, 50 mm for step 2, and 200 mm for step 3. Based on the reaction times for steps 1 and 2 on chip 1, **1** and the ^18^F-labeling reagent in DMSO were run into inlets **F** and **G** at a flow rate of 0.56 μL/min for [^18^F]fluorination, respectively (Fig 10a). At 5 min after the beginning of the reaction, TPAH was run into inlet **I** at a flow rate of 1.12 μL/min, and after another 30 s, TSTU was run into inlet **K** at 2.24 μL/min. DMSO/water (90/10) was introduced into the reaction stop reagent injection port **N** to stop the reaction. Thus, the reaction times were 5 min for ^18^F-fluorination, 30 s for hydrolysis, and 1 min for succinimidylation. Chip 2 was placed on an aluminum chip holder equipped with a built-in ceramic heater to perform reactions at a predetermined temperature. When the microreactor was heated, the surface temperature of chip 2 was measured using a thermocouple and thermography. Chip 2 was heated to a uniform temperature (± 5°C) to set the temperature (see Electronic Supporting Information). The three-step reaction for [^18^F]SFB on chip 2 was successfully completed within 6.5 min of residence time. The conversion yield was 64 ± 2% (*n* = 5); the yield rate and reaction efficiency are substantially better than those of the one-pot synthetic method [reaction time 17 min, 48 ± 9% (*n* = 6) conversion yield]. These results show that the use of the developed microreactor gave a higher radiochemical yield of [^18^F]SFB with a shorter reaction time than the use of the one-pot synthetic method.

**Fig 11:**
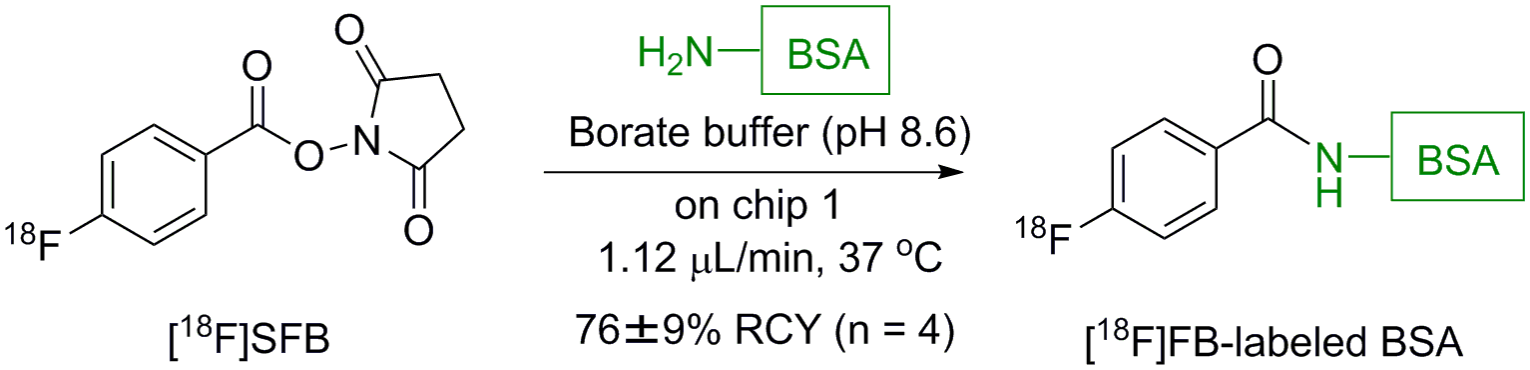
Radiolabeling of BSA with [^18^F]SFB synthesized using chip 2 (data are the mean ± S.D., *n* = 4).

To evaluate the quality of the [^18^F]SFB synthesized by using chip 2, the reaction mixture obtained from outlet M was purified by solid phase extraction, as done for the above one-pot synthesis, and applied for radiolabeling with bovine serum albumin (BSA) (Fig 11). 4-[^18^F]Fluorobenzoyl ([^18^F]FB)-labeled BSA was obtained in good conversion yields (76 ± 9% from [^18^F]SFB, *n* = 4), which indicated that the quality of [^18^F]SFB synthesized using chip 2 was equal to that synthesized using conventional methods.

## Conclusions

We have demonstrated the feasibility of a new continuous-flow synthetic method for a multistep procedure using a single microfluidic chip. Continuous-flow synthesis of [^18^F]SFB using chip 2 gave a higher conversion yield and a shorter reaction time than that using a batch reactor. In our microreactor, the reaction efficiency was improved by enabling the reaction to be completed rapidly. After the preparation of reagents injected to the microreactor, the synthesis was automatically performed by supplying the reagents continuously and reproducibily achieved. In addition, this microreactor can be operated easily and does not require advanced skills. Continuous-flow synthetic procedure using this microreactor should therefore be readily adaptable to the automated production of [^18^F]SFB. The re-design of the microfluidic chip to enable the treatment of large quantities of reaction solution in a short time is required for commercial application.

## Supporting Information

S1 Fig Temperature distribution on chip 1. (a) Microreactor device, (b) chip design and temperature measurement points, (c) point temperatures recorded using digital thermometer, and (d) thermographic images. The results demonstrated that chip 1 was uniformly heated together within ±5°C of the set temperature.
(TIF)

S2 Fig Microscopy images of channels (solvent: DMSO, temperature: 120°C, retention time: 10 s). It was observed that the solution has been thoroughly mixed at a point III where is after 5 s from the intersection of the two solutions (point I).
(TIF)

S3 Fig Temperature distribution on chip 2. (a) Microreactor device, (b) chip design and temperature measurement points, (c) point temperatures recorded using digital thermometer, and (d) thermographic images. The results demonstrated that chip 1 was uniformly heated together within ±5°C of the set temperature.
(TIF)
