# Supplementary figures and images for: Continuous-Flow Synthesis of N-Succinimidyl 4-[18F]fluorobenzoate Using a Single Microfluidic Chip
Source: PLoS One. 2016 Jul 13;11(7):e0159303. doi: 10.1371/journal.pone.0159303 (PMC4943714; doi:10.1371/journal.pone.0159303)

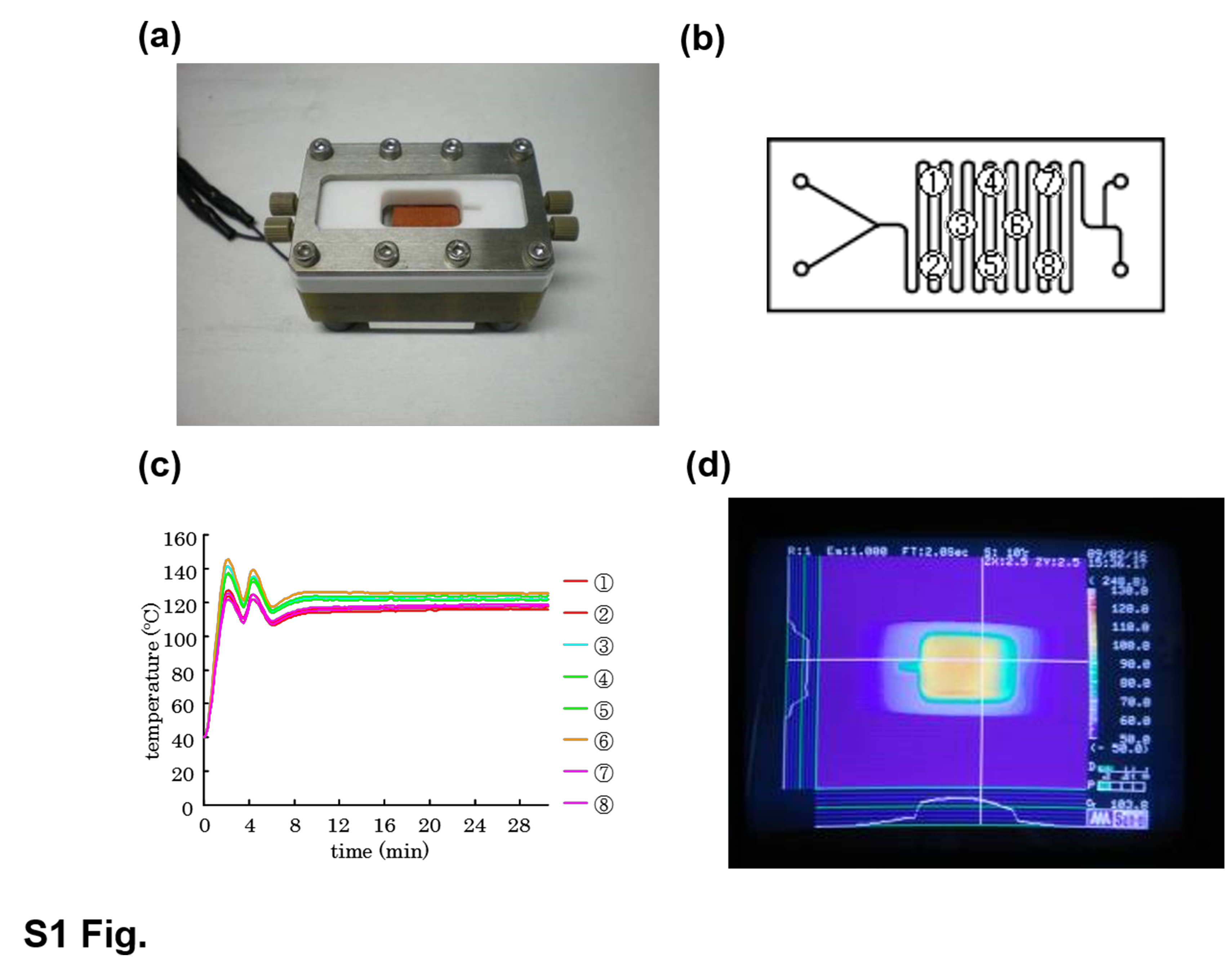

Supplement: S1 Fig — (a) Microreactor device, (b) chip design and temperature measurement points, (c) point temperatures recorded using digital thermometer, and (d) thermographic images. The results demonstrated that chip 1 was uniformly heated together within ±5°C of the set temperature. (TIF) [file pone.0159303.s001.tif]

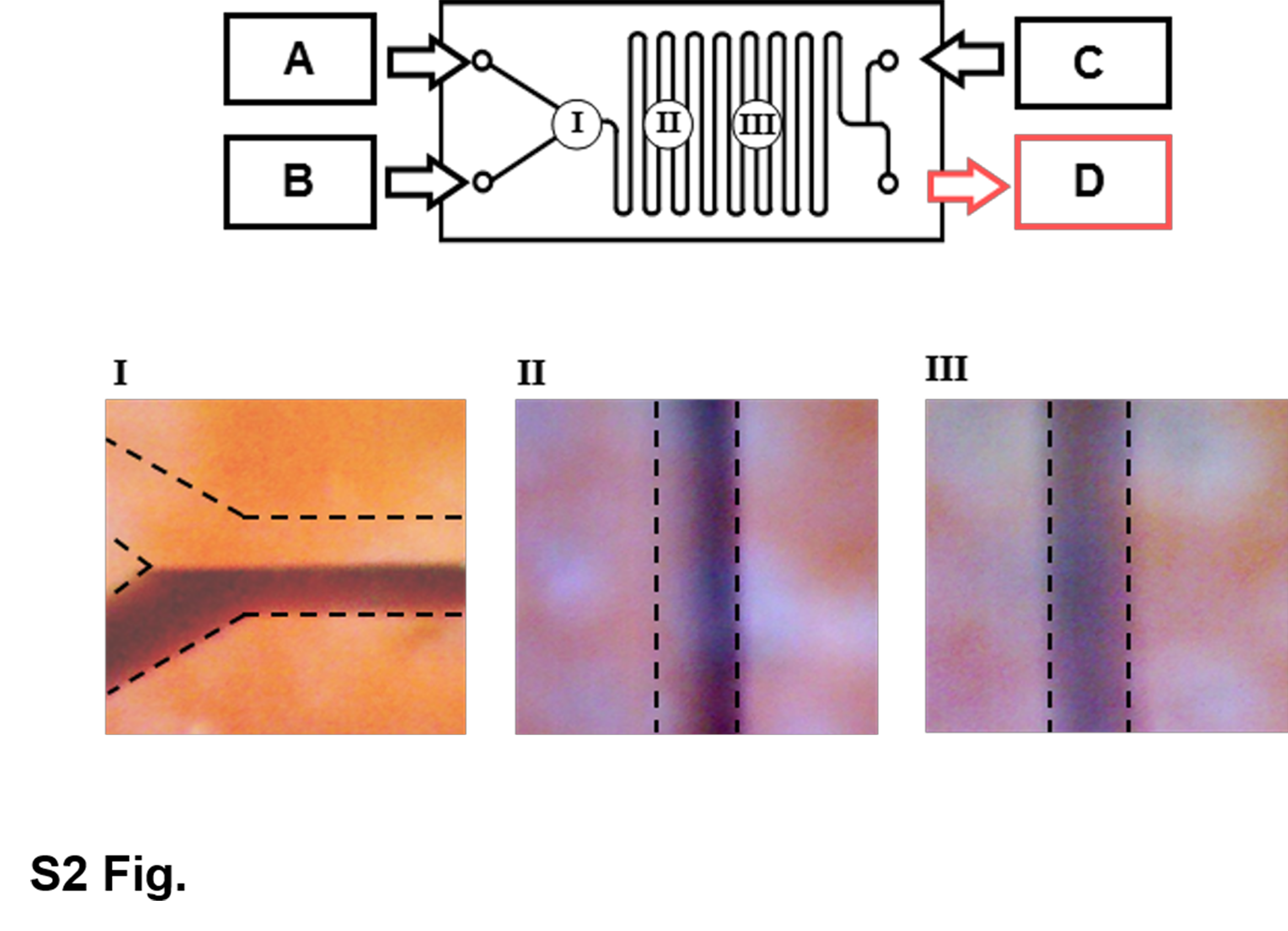

Supplement: S2 Fig — It was observed that the solution has been thoroughly mixed at a point III where is after 5 s from the intersection of the two solutions (point I). (TIF) [file pone.0159303.s002.tif]

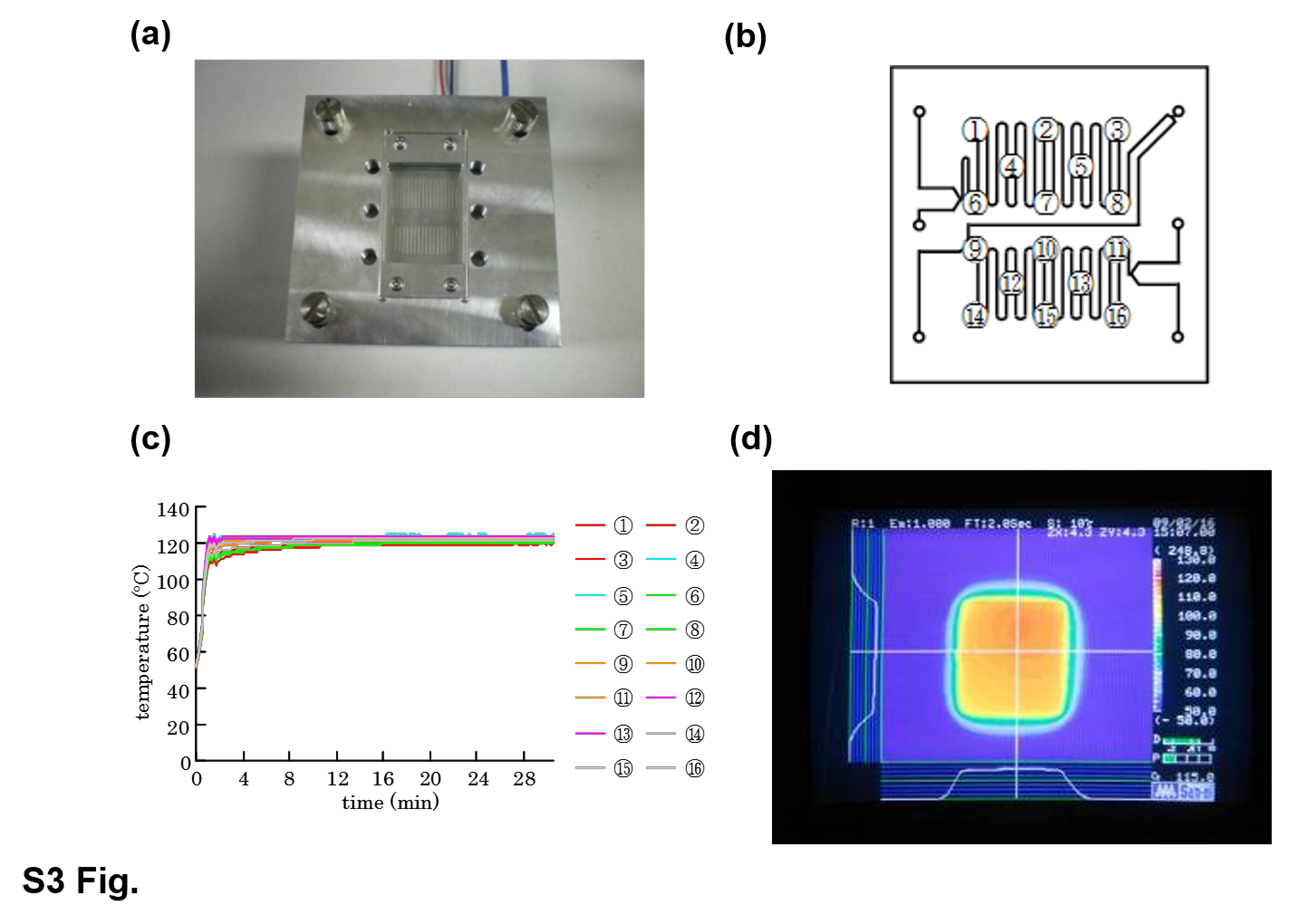

Supplement: S3 Fig — (a) Microreactor device, (b) chip design and temperature measurement points, (c) point temperatures recorded using digital thermometer, and (d) thermographic images. The results demonstrated that chip 1 was uniformly heated together within ±5°C of the set temperature. (TIF) [file pone.0159303.s003.tif]
